# Dietary Influence on Body Fluid Acid-Base and Volume Balance: The Deleterious “Norm” Furthers and Cloaks Subclinical Pathophysiology

**DOI:** 10.3390/nu10060778

**Published:** 2018-06-16

**Authors:** Qi Qian

**Affiliations:** Division of Nephrology and Hypertension, Department of Medicine, Mayo Clinic, School of Medicine, Rochester, MN 55905, USA; qian.qi@mayo.edu; Tel.: +1-(507)-266-7960

**Keywords:** dietary salt, hydration, dietary protein, acidosis, disease prevention

## Abstract

The popular modern diet, characterized by an excess of animal protein and salt but insufficient in fruits, vegetables and water, is a poor fit for human physiological and homeostatic regulatory systems. Sustained net acid and sodium retention, coupled with an insufficient intake of cardiovascular protective potassium-rich foods and hydration in the modern diet can give rise to debilitating chronic organ dysfunction and ultimately, mortality. This holds true, especially in our aging population who are already facing inevitable decline in organ functional reserve. Importantly, in most cases, despite the mismatch and adverse effects to multiple organ systems, plasma electrolyte and acid-base parameters can, on the surface, be maintained within a “normal” reference range, primarily by activating (often maximally activating) compensatory homeostatic mechanisms. These diet-induced effects can thus be clinically silent for decades. Embodied in the chronic corrective homeostatic processes, however, are real risks for multiorgan damage. According to the Dietary Guideline Advisory Committee (DGAC), half of American adults have one or more chronic diseases that are preventable with dietary modification. Here, homeostasis of body fluid acid-base, sodium, potassium and water is examined. Our current dietary habits and their required regulatory adaptation, maladaptation and relevant physiology and pathophysiology are discussed. A framework of dietary modifications to avoid a propensity for maladaptation and thus lowers the risks of common modern diseases (primary prevention) and minimizes the risk of chronic and age-related disease progression (secondary prevention) is emphasized. Although there are other variables at play, a key to restoring the all-important dietary potassium to sodium ratio is greater consumption of vegetables/fruits and adopting salt temperance. Dietary and nutritional optimization is an under-emphasized area of health care that has an enormous potential to temper the epidemics of prevalent chronic diseases in modern society and improve population health.

## 1. Introduction

Evidence has been mounting that the current nutrient profile of our modern diet requires extreme homeostatic regulation and is, in the long run, detrimental to many aspects of host physiology and give rise to risks of multiple chronic diseases. According to the Dietary Guideline Advisory Committee (DGAC), half of all American adults, ~117 million individuals, have one or more chronic diseases that are preventable with dietary improvement [1]. Such diet-induced health problems are more impactful in an era of aging populations, showing a reduced capacity to adapt due to an age-related decline in metabolic capacity and organ functional reserve. This situation largely accounts for the increased prevalence of many chronic diseases seen with aging, including hypertension (HTN), atherosclerotic vascular diseases and chronic kidney disease (CKD). Said differently, poor diets are the greatest contributor to the high burden of chronic diseases in the United States. Currently, only 1% of Americans satisfy the criteria for ideal cardiovascular health, 46% have hypertension, about 50% have either prediabetes or diabetes, and about 14% have CKD. Modifications of nutrient intake in the modern diet can minimize the risks of maladaptation, including acid accumulation, salt overload, deficiency of potassium (K^+^) and fiber and under hydration; such modifications can positively impact general health and are of significant importance. This report examines physiological regulations of electrolytes and acid-base influenced by dietary intake and the relevant maladaptations that stem from a suboptimal diet. It is intended to provide an assessment of the pathophysiological nature of the popular contemporary “normal” diet and thereby the likelihood of potential benefits with instituting a more balanced diet that better fits the body’s physiological needs.

## 2. Changes in Key Dietary Nutrient Compositions Since the Ancestral and Premodern Diet

**Figure 1:**
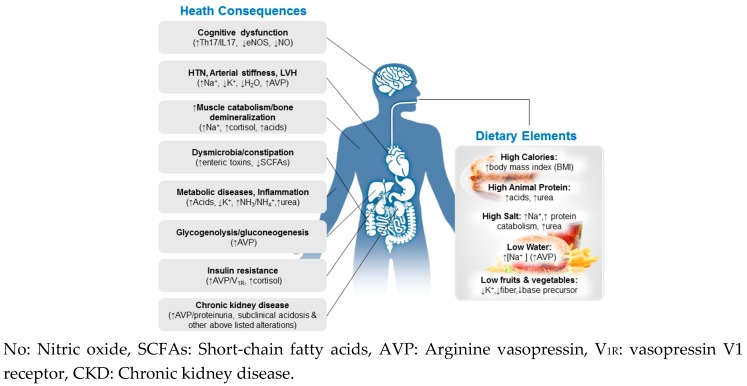
Multisystemic effects of the modern diet, characterized by high calorie, animal protein, and salt but insufficient water, fruits and vegetables (**Right**). Key alterations in multiple organ systems (**Left**) may be elicited by the modern diet.

The human body’s nutritional requirements are derived from natural selection over millions of years of evolution and dietary exposure of humans (*Homo sapiens*) to wild animals and uncultivated plant foods. The relatively recent (~10 thousand years) agricultural and industrial transition has resulted in a sharp turn in the human diet from his ancestral diet of net-neutral or base-generating to a modern diet (typified by an average American diet [2]) of net-acid generating (high intake of animal protein), from low salt (~1–3 gm/day) to high salt (10–15 gm/day), from high K^+^ and high fiber to low K^+^ and low fiber, along with insufficient water intake [2,3,4], high energy (kcal) and cholesterol. From Paleolithic times to the present, the ratio of dietary K^+^/sodium (Na^+^) has decreased by a factor of ~25, reflecting a marked K^+^ “deficiency” and Na^+^ surfeit in relation to our metabolic design; fiber intake has fallen from a high of ~100 to 11–15 gm/day. These drastic dietary deviations are not natural to the human body and require constant activation of multi-systemic homeostatic mechanisms to temper the risk of imbalance and its health consequences (Figure 1). Moreover, the human body undergoes dynamic changes throughout its lifespan. With aging, metabolic and organ reserves universally decline. A diet that might be tolerated by a young adult would not be appropriate for the same person several decades later and could thus be disease-causing. Failure to adjust diet and lifestyle accordingly can give rise to maladaptation (or inability of the body to compensate), leading to the development of a number of chronic diseases, commonly seen in the elderly, including hypertension (HTN), diabetes, cardiovascular diseases and CKD.

## 3. Role of the Kidneys in the Active Maintenance of Body-Fluid Balance

The kidneys are the main organs in the regulation and maintenance of body-fluid composition and balance. Acid-base, salt and water regulation comprise the major body fluid components subjected to active kidney-mediated regulations. The kidney workload can be heavily influenced by dietary intake.

### 3.1. Acid-Base and Potassium Balance

Consuming a typical modern diet, an adult generates ~0.7–1 mEq/kg body weight of net non-volatile (non-carbonic) acids (~50 to >70 mEq/day) [5], also known as the daily net endogenous acid production (NEAP). To achieve a balance, the kidneys have to remove the net acids, through the process of renal net acid excretion (RNAE). When in balance, NEAP is expected to equal RNAE. The kidneys accomplish this balance by saving (a reclamation process) all filtered HCO_3_^−^ through glomeruli and generating new HCO_3_^−^ via excreting extra acids (to offset a net positive NEAP). Specifically, the kidneys: (1) reclaim ~4500 mmoL of filtered HCO_3_^−^ daily; and (2) regulate the excretion of urinary titratable acids (TAs) and the generation and excretion of ammonia/ammonium (NH_3_/NH_4_^+^) according to the amount of positive NEAP. TA excretion is responsible for ~1/3 of RNAE. It is a relatively fixed and low-capacity system, limited by dietary phosphate/phosphate-precursor intake and glomerular phosphate (HPO_4_^2−^) filtration. The remaining ~2/3 of RNAE are accomplished via renal generation and excretion of NH_3_/NH_4_^+^. For every NH_4_^+^ excreted from urine, equimolar HCO_3_^−^ is gained back to the body. The renal NH_3_/NH_4_^+^ genesis-excretion system is a high-capacity acid-excretion system. In settings of heavy acid load, its acid-excretion capacity can rise nearly 10-fold, from ~30–40 mmoL/day to >200 mmoL/day. Thus, RNAE can be estimated numerically by calculating urine excretion of NH_4_^+^ + TA − HCO_3_^−^. Acidemia and hypokalemia, even at mild degrees, can stimulate NH_3_ genesis, while alkalemia and hyperkalemia prompt the opposite effect.

A low dietary K^+^ intake (less than the recommended 4700 mg, or ~120 mmoL/day [6]) is typically due to a reduced consumption of plant-foods (fruits and vegetables), notably insufficient in the general population [1]. The modern diet has a K^+^/Na^+^ ratio of ~0.4 whereas in a premodern era, the ratio was almost always >3 and often near 10. As plant foods are also a rich source of base or base-equivalents and fiber, a low K^+^ diet is coupled with low base and low fiber, causing a net acid accumulation (positive NEAP) and sluggish bowel transit from lacking fiber, which can alter gut microbiota [7].

NEAP is largely determined by the sum of dietary intake of acid and base precursors. With dietary modifications, NEAP can be converted from a net-acid (positive NEAP) to a net-base generating (negative NEAP). Indeed, in a group of healthy athletic adults, baseline positive NEAP can be modified to a net-neutral or base-generating (negative NAEP) by adding dietary fruits and vegetables without changing meat consumption [8].

NEAP has traditionally been quantified based on a detailed dietary inventory. The Remer-Manz method is one of the established methods for calculating NEAP. It calculates the potential renal acid load (PRAL), which estimates the acidic potential of foods [9]; it is, however, too cumbersome to be routinely used in practice. Frassetto et al. [10] found that a large degree of acid-predictive power in the Remer-Manz model resides in the amount of protein and K^+^ content of a diet, as proteins, especially of animal origin, are acid-procurers, and K^+^, largely coupled with base-equivalents, represents base-precursors. Thus, the dietary protein (g) to K^+^ (mM) ratio can nicely reflect PRAL and is highly correlated with RNAE and thus NEAP.

**Figure 2:**
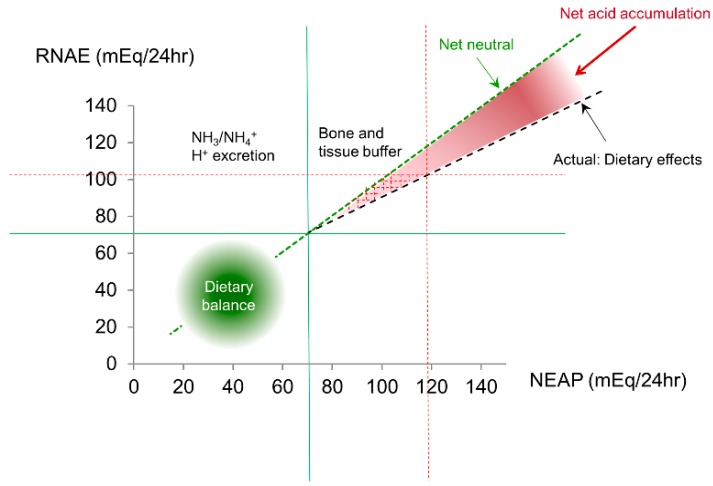
NEAP is largely determined by dietary intake. When NEAP is <~70 mEq/L, there will be minimum net acid accumulation. However, when a diet generating a large NEAP (>70–75 mEq/day), RNAE can become insufficient to match the large quantity of NEAP, resulting in a surplus of acids that have to be neutralized by mechanisms other than the kidneys (mainly bones). The red dotted lines give an example of NEAP (~120 mEq) and RNAE (~100 mEq) mismatch.

It should be noted that certain plant foods, when processed and refined, could generate acids, contributing to NEAP elevation. These included processed cereal-grains (wheat, rice, rye, oats, barley, corn, and sorghum); if average breakfast cereal-grain energy in the modern diet were replaced with nuts, beans, vegetables, seeds and a variety of fruits, our NAEP would move substantially towards less positive (less acids) [11]. It is also important to recognize that, even in healthy adults, a net acid (H^+^ ion) accumulation in the body can occur when NEAP exceeds ~70 mEq/day [5] (Figure 2). Cellular H^+^ accumulation reduces HCO_3_^−^, which, in turn, alters soluble adenylyl cyclase (sAC) activity [12] and can thus alter the production of cAMP, a ubiquitous second messenger. Persistent H^+^ accumulation can, therefore, affect a variety of biological systems and engender adverse consequences; some of these are detailed below.

Potassium (K^+^) is the dominant intracellular cation, with >98% of total body K^+^ (~3500 mmoL) being distributed in the intracellular compartment and <2% (~65–70 mmoL) in the extracellular fluids. The sharp intracellular to extracellular K^+^ gradient is necessary to maintain the plasma membrane potential, critical for the functions of multiple tissues, especially the heart, nerves and skeletal muscle. K^+^ also participates in the regulation of cell volume and cellular pH. The kidneys are the major K^+^ regularly organ. K^+^ is freely filtered through glomeruli. The proximal tubules reabsorb ~65% of the filtered K^+^, while the thick ascending limb of the loop of Henle reabsorbs ~25%. The distal nephron (the distal convoluted tubule and collecting duct) is the major site of renal K^+^ regulation. Depending on physiological needs, the distal nephron can either excrete or absorb K^+^. Key factors that promote distal nephron K^+^ excretion include serum K^+^ elevation, increased distal tubular Na^+^ delivery, increased rate of tubular fluid flow, increased serum aldosterone level and alkalosis [13].

### 3.2. Salt and Water Balance

The regulations of salt and water are tightly inter-connected. Serum Na^+^ concentration represents water balance and is the primary determinant of serum osmolality. In the kidney, arginine vasopressin (AVP) and tonicity enhancer element binding protein (TonEBP, also known as transcription factor nuclear factor of activated T-cells 5, NFAT5) independently and synergistically regulate water absorption, maintaining serum Na^+^ concentration in the range of 135–145 mEq/L. In addition to water regulation, AVP also retains Na^+^, thus influencing volume balance.

AVP binds to V2 receptors in the basolateral membrane of collecting ducts, activates adenylyl cyclase-mediated cAMP generation and PKA-mediated signaling pathways, leading to phosphorylation and the apical membrane insertion of aquaporin 2 (AQP2) water channels. This, in turn, leads to free water absorption from the tubular lumen to the circulation, driven by the presence of a tubulomedullary osmotic gradient. TonEBP, responding to increasing extracellular interstitial tonicity, increases transcription of genes involved in the synthesis and transport of organic osmolytes [14]. When activated, both AVP and TonEBP increase AQP2 gene expression and apical membrane AQP2 distribution.

On a high salt diet (≥12 gm/day), healthy adults are able to raise the amount of urine Na^+^ excretion, typically without triggering extra water intake (lacking thirst stimulation) [15]. The kidneys are responsible for excreting concentrated Na^+^ and simultaneously conserving water. This process requires the kidney to absorb extra Na^+^ and urea from tubular fluids to raise intramedullary hypertonicity in order to facilitate water absorption/conservation. Indeed, recent studies show that a high salt diet activates an array of metabolic processes, including enhanced hepatic uricogenesis and muscle catabolism through, at least in part, activating glucocorticoid receptors [15,16]. Also seen in association with a high salt diet is elevation of circulating AVP. AVP is known to cause intraglomerular hypertension, mesangial contraction and proliferation and intra-renal activation of the renin-angiotensin-aldosterone system (RAAS) [17] (Qian, 2018 in press). When persistent and habitual, these homeostatic adaptive processes can become maladaptive. Accordingly, a recent population-based study shows that plasma copeptin (a surrogate marker of AVP) is a predictor for the occurrence of CKD [18], a chronic disease affecting ~13.4% of the population worldwide [19]. With the advent of Na^+^ imaging NMR technique [20], a volume-independent pool of Na^+^ in the skin and muscle has been revealed; heavy epidermal and dermal Na^+^ signal is associated with refractory HTN and risk for cardiac hypertrophy [21,22].

## 4. Maladaptations to the Modern Diet and Health Implications

### 4.1. Net Acid Retention

The average American consumes 1.2 gm protein/kg/day [23], exceeding the recommended intake (~0.8–1.0 gm/kg/day) for a healthy adult. A large proportion of ingested protein are animal proteins that are acid-producing. It is conceivable that the acid-base homeostatic mechanisms are activated in most individuals to prevent acidosis. Indeed, maladaptive net acid retention (low-grade and subclinical acidosis) [5], although subtle, is more common than recognized. Such habitual diet-related acid retention can be consequential, especially for vulnerable populations such as the elderly with declining kidney functional reserve [24] –check- and patients with CKD. That said, one should not discount the critical importance of adequate amounts and quality of dietary protein intake, given its known contribution to the maintenance of muscle, bone and multiple organ growth and metabolic regulatory processes.

Wesson et al. [25] recently reported their finding of a net positive acid retention in 2/3 nephrectomized rats but not in sham-operated rats fed with the same chow. The degree of acid retention in 2/3 nephrectomized rats increases proportionally with acidogenic and acidic diet (casein and NH_4_Cl). The diet-induced acid retention was highly associated with urinary markers of kidney tubulointerstitial injury and bone matrix injury. Remarkably, the acid retention and its injurious effects could all be present without an overt systemic metabolic acidosis (reductions of serum HCO_3_^−^ were subtle and had remained within the reference range). The retained acids seem largely buffered by the bones. The underlying mechanisms of kidney and bone injury induced by acid-retention can be multiple, including NH_3_/NH_4_^+^ genesis, inflammation and activation of complement and RAAS [26,27,28]. Similar correlations of acid retention without overt acidosis has also been observed in patients with reduced kidney function [27].

In two large population-based studies from community-dwelling elderly Koreans (KoGES), increasing NEAP and low-normal HCO_3_^−^ is associated with subclinical acidosis and CKD [29,30]. Consistently, acid loading in a number of studies has been shown to be associated with proteinuria, progressive reduction of eGFR and development of CKD [31,32,33,34]. In addition, mild reduction of serum HCO_3_^−^, signifying acidosis, in the general population is associated with all-cause, cardiovascular and cancer mortality [35,36].

Oral salt intake in individuals with compromised kidney clearance, as in CKD patients, contributes to acid generation. It has long been known that intravenous infusion of 0.9% NaCl (saline) can have very different effects on the body’s salt-load and acid-base balance compared to oral salt intake in adults without CKD. Oral intake evokes a robust Na^+^ unloading in the gut and urine (coupled to Cl^−^ wasting) while intravenous saline infusion of the same amount will inevitably cause Na^+^ and Cl^−^ retention, leading to volume expansion and hyperchloremic acidosis [37]. This difference, termed the gut-renal axis, has been investigated to a reasonable extent. A combined role of uroguanylin and pendrin is indicated in this process [38,39]. Oral salt ingestion stimulates gut urogranulin secretion through a guanylate cyclase C signaling pathway, which leads to cGMP-dependent inhibition of Na^+^/H^+^ exchange and interferes with gut salt absorption [40]. Uroguanylin is also expressed robustly in the kidneys, where it inhibits NHE (Na^+^-proton exchanger) and inhibits phosphodiesterase 3 in the proximal tubules that enhance cyclic AMP/PKA-induced Cl^−^ and HCO_3_^−^ exchange. With these actions of uroguanylin, the proximal tubular salt absorption is markedly blunted. In the collecting duct, uroguanylin inhibits ROMK (renal outer medullary potassium channel) via inhibiting arachidonic acids [40]. Uroguanylin also inhibits pendrin expression in the intercalated cells [41] and thus inhibits collecting duct pendrin-mediated Cl^−^ absorption, (in exchange for HCO_3_^−^). HCO_3_^−^ is known to stimulate ENaC (epithelial sodium channels) in the collecting ducts [39]. With diminished HCO_3_^−^ secretion by pendrin, Na^+^ absorption via ENaC is diminished. Together, NaCl absorption in the collecting ducts is markedly reduced by the joined effects of oral salt ingestion on uroguanylin and pendrin. In patients with compromised GFR and renal tubular dysfunction, as in CKD, despite the marked elevation of gut-source uroguanylin, renal NaCl unloading is impaired, resulting in volume expansion and hyperchloremic acidosis.

Although subtle and to a much smaller degree, such volume and hyperchloremic effects of oral salt loading have been described in a study of a cohort of healthy individuals (*n* = 77) by Frassetto et al. [42]. Hyperchloremic acidosis is associated with the lowest tertile of creatinine clearance, 73 ± 11 mL/min, but not in those with the highest tertile, 125 ± 16 mL/min. The reduction of serum HCO_3_^−^ in those with hyperchloremia was mild and remained within the reference range. Thus, even in healthy adults, without a categorical diagnosis of CKD, heavy dietary salt could elicit acidosis, which has been associated with multitude adverse effects [37]. Diet-induced acid retention appears common in children. Studies have demonstrated that, even in a mild degree (serum HCO_3_^−^ remains within reference ranges) a low grade metabolic acidosis induced by a typical modern diet in children can trigger elevated glucocorticoid production and urinary excretion of glucocorticoid products, potentially contributing to muscle catabolism and impaired bone growth [43,44]. Subclinical acidosis can also induce growth hormone/insulin-like growth hormone-1 (GH/IGF-1) insensitivity [45]. Diminished serum IGF-1 in patients with renal failure has been linked to a higher rate of mortality [46]. In addition, acidosis has been linked to altered insulin sensitivity [47], skeletal demineralization, nephrolithiasis, and, in hypertensive adults, cognitive impairment [48]. These observations further emphasize the critical role of active kidney compensation to defend the systemic acid-base balance in the setting of the acidogenic diet.

### 4.2. Inadequate Dietary Potassium (K^+^)

The modern diet lacks sufficient K^+^ [2]. A low or low-normal serum or plasma K^+^ concentration, a consequence of insufficient intake, can stimulate kidney NH_3_/NH^4+^ genesis and RAAS activation, contributing to systemic inflammation, protein catabolism, development of CKD and, in patients with CKD, promoting CKD progression [49]. Kaluretic diuretics, commonly used for hypertensive and CKD patients, can further exacerbate K^+^ deficiency and its adverse effects [50]. Abundance of dietary K^+^ is epidemiologically associated with a reduction in HTN, stroke and other cardiovascular diseases [51].

Potassium has been shown to reduce vascular smooth muscle cell proliferation and migration. It also reduces neointimal formation following vessel injury. These effects were implied in a population-based study, showing an association between impaired proliferation of vascular smooth muscle cells and reduced monocyte adherence to the vessel walls [52]. Recently, Sun et al. [53] demonstrated a causal role for dietary K^+^ in the regulation of osteogenic differentiation and calcification of vascular smooth muscle cells, both in vitro and in atherosclerotic animal models. Specifically, lower levels of extracellular fluid K^+^ induce vascular smooth muscle cell osteogenic transformation by elevating intracellular calcium. The latter activates CREB (cyclic AMP response element-binding protein) leading to an enhanced expression of osteogenic markers, e.g., RUNX-2, and simultaneously reduced smooth muscle cell markers, e.g., α-actin. Remarkably, even a slight serum K^+^ reduction (mean K^+^ level, 3.70 ± 0.21 mEq/L) in mice can trigger significant vessel calcification associated with elevated pulse-wave velocity, a reliable indicator of aortic stiffness. On the contrary, when K^+^ levels are raised to ~4.73 mEq/L by dietary modification, signs of osteogenic differentiation were abrogated, and vascular calcification prevented. Consistent with the notion of K^+^ being protective to vasculature, a high ratio of urine Na^+^/K^+^ excretion (indicative of high Na and low K^+^ intake) has recently been linked to the genesis of HTN [54].

A risk of developing CKD associated with insufficient K^+^ intake has recently been shown in a prospective, population-based study (PREVED), involving 5215 adults (age, 28–75 years) with a median follow-up of 10.3 years [55]. CKD risk has also been shown to increase significantly with a reduced urine K^+^ excretion [55], signifying a low K^+^ diet. For each 21 mmoL decrement of daily urine K^+^ excretion, there was a 16% higher risk of developing CKD (defined as eGFR <60 mL/min/1.73 m^2^ or albuminuria >30 mg/24 h or both) over the follow-up period. This is consistent with a prior cross sectional study of dietary K^+^ intake [56] and urine K^+^ excretion on the risk of CKD initiation and progression [57,58]. Even in dialysis patients, low K^+^ is associated with a significant increase in mortality [59,60].

### 4.3. Salt Overconsumption and Insufficient Hydration

Overconsumption of salt, a major dietary deviation in the general population [2,61], is often coupled to an inadequate water intake [3]. Such a combination raises serum osmolality which stimulates AVP secretion [16]. AVP has been shown to induce glomerular hyperfiltration and proteinuria, both of which increase the risk of CKD [62] and mortality [63,64]. Circulating copeptin is a surrogate marker for AVP; its elevation has repeatedly been associated with the development of proteinuria, especially in the elderly, and the development of CKD [65,66]. These maladaptive effects, coupled with excess dietary acids can be synergistically detrimental to kidney function. A high salt diet is also well known to raise body fluid volume, which elevates type-B natriuretic peptide (BNP). Recent studies have shown that a subtle volume increase, shown as BNP elevation, can independently raise mortality even in patients without evidence of heart failure [67,68].

A high salt diet can incite abnormalities above and beyond body fluids and acid-base effects. Both AVP (copeptin) and a high-salt diet can stimulate glucocorticoid secretion, causing muscle catabolism and hepatic urea generation [15,16,69]. AVP can alter hepatic glycogenolysis and gluconeogenesis as well as cause impaired insulin secretion leading to insulin resistance and hyperglycemia. In the gut, high-salt challenges in normal adults diminishes intestinal *Lactobacillus* species. The reduction of metabolites from the gut *Lactobacillus* species, especially indole-3-lactic acid (ILA), can compromise IL22 production. IL22 is a member of the IL10 family of cytokines; it increases production of mucin-associated proteins by the gut epithelium and increases mucosa resistance against infection [70]. Lacking ILA from a high-salt diet has also been shown to activate the gut immune system, increasing circulation TH17 cells and causing salt-sensitive hypertension [71]. Moreover, a high salt diet, through inducing gut TH17 activation, has recently been shown to impair cognition. Circulatory interleukin 17 (IL-17), through ROCK-mediated inhibitory eNOS phosphorylation, reduces endothelial nitric-oxide (NO) production, causing a vasodilatory defect of microvasculature and brain micro hypoxia, leading ultimately to cognitive dysfunction, especially impaired global and executive decision-making capacity [72].

Chronic low-grade under-hydration has been associated with impaired skilled performance, reduced cognition, and signs of perceived fatigue and lack of energy [73]. Under-hydration can also render individuals vulnerable to suboptimal adaptation or outright maladaptation, inciting kidney dysfunction and other health complications in times of physical stress or exposure to a hot climate. It is not surprising that chronic under-hydration has also been linked to nephrolithiasis [74] and risk of metabolic syndrome [75] and CKD [76]. In a 2018 report by Enhorning et al. [18], elevated serum copeptin (reflecting a high AVP state) in two population-based cohorts with follow ups of 8.7 and 19.6 years effectively predicted the risk of CKD development.

### 4.4. Excess Urea Production and Metabolism

A modern protein-rich diet increases protein fermentation in the intestinal tract, causing the generation of multiple potentially toxic metabolites. In patients with compromised kidney function, these metabolites can accumulate and contribute to uremia [77,78,79]. It is well known that urea, a metabolic product of protein/amino acid and nitrogen metabolisms, is produced in excess when exposed to a high-protein and high-salt diet [16]. Excessive urea generation also requires more urine volume to excrete. As with adequate protein intake, urea generation is important for multiple physiological functions including urine concentration; sufficient kidney function typically guarantees urea excretion to maintain a net balance. In the setting of compromised kidney clearance and under hydration, urea can accumulate in the circulation. Contrary to the historical impression of urea being an inert molecule, mounting evidence indicates that, in fact, urea is metabolically active. In body fluids, it is equilibrated to a small amount of cyanate. The latter is further converted to isocyanate, which is capable of carbamylating multiple molecules causing insulin resistance, systemic inflammation, and endothelial dysfunction that all can negatively impact cardiovascular outcome and mortality (reviewed in [80]). When diffused into the gut lumen, urea can be converted to ammonia (NH_3_) by intestinal urease-producing bacteria. Such bacteria could be induced to grow and flourish in the gut by high protein intake or by sustained urea exposure. NH_4_OH, derived from gut NH_3_, predictably raises intestinal pH, which exerts corrosive effects on the junctions between gut epithelial cells and compromises the integrity of intestinal barrier. Studies have shown the disruption of intestinal epithelial tight-junctions and diminished expression of tight-junctional proteins can cause gut bacterium translocation, immunoactivation and systemic inflammation [81,82]. Intact bacteria have been isolated in atherosclerotic plaques, as well as in a number of organ systems. A low fiber diet, typically associated with a high protein diet [8], can further amplify the urea-mediated detrimental effects by reducing gut transit, promoting dysbiosis (diminished polysaccharide-fermenting bacteria) that reduces the formation of gut-nurturing short chain fatty acids (SCFAs: mainly acetate, butyrate and propionate) from undigested fibers. Notably, SCFAs, especially butyrate, are markedly diminished in patients with hypertension [83], consistent with the role of inflammation from dysbiosis in the genesis of HTN [84]. SCFAs, through olfactory receptor 78 (OLFr78) and G-protein coupled receptor 41 (Gpr41), also reduce renin generation and vascular resistance [85]. Dysbiosis has also been associated with nephrolithiasis [86], genesis and progression of CKD and poor cardiovascular outcomes [49,87].

## 5. Prevention of Acid-Base and Electrolyte Maladaptive Responses, the Power of Dietary Modification

Studies have shown that diet-related detrimental effects can fortunately be minimized by dietary modification, specifically, reducing the over-consumption of salt and animal-foods and commensurately increasing unrefined plant foods and hydration.

Increasing dietary fruits and vegetables increases both base/base equivalents and K^+^ in body fluids and has been associated with reduced blood pressure and lowered risk of cardiovascular diseases including heart failure [88,89] and mortality [90,91,92,93]. Interventional studies have shown that a more abundant dietary K^+^ intake lowers systolic and diastolic blood pressure [94,95]. KHCO_3_ supplementation also attenuates urinary nitrogen [96,97] and calcium excretion [98] and is associated with a higher circulating IGF-1 level.

In CKD patients with metabolic acidosis, base administration slows CKD progression and reduces mortality [8]. Proteins from animal foods, compared to proteins from plant (soy) proteins, are significantly more acidogenic and are potent inducers of renal hyperfiltration [99], which is a known risk factor for proteinuria and CKD [62]. A good supportive example is the recent population study from Singapore (the Singapore Chinese Health Study, *n* = 63,257), which showed a lower risk of renal failure in those adhering to a diet rich in soy and legumes than those with diets heavy in red meats [100]. In two randomized interventional trials, correcting low-grade metabolic acidosis with oral alkaline administration in children abrogated elevated activity of glucocorticoids [101] seen with a high salt and acidogenic diet, thus avoiding the pro-catabolic effects of glucocorticoids. These findings are consistent with the notion of minimizing dietary acid and salt excess in health promotion.

With regard to inadequate hydration, another common dietary deficiency in the general population [3,4], several interventional studies in children have consistently shown that added water intake enhances cognition and schoolwork performances [102,103,104]. In adults, greater water intake, demonstrated as low-normal range of serum osmolality (less copeptin elevation) has clearly been associated with a lower occurrence rate of CKD [18,105]. In a 2018 multicenter interventional study of patients (*n* = 631) with CKD stage 3 and proteinuria, a one-year increase in self-reported daily fluid intake by 0.6 to 0.8 L resulted in a small but significant reduction in serum copeptin (−2.2 pmoL/L), associated with a significantly less l--r decline in serum creatinine clearance (no significant change in the eGFR [estimated glomerular filtration rate]) [106].

Although there can be no question that there are biological/physiological boundaries to human body homeostatic regulation, to what extent dietary modifications can maximize health and minimize hazards needs further detailed and quantitative investigation. Given the emerging data, it seems sensible to modify our dietary behavior so that homeostatic and adaptation systems can operate well within the boundaries of our biological limits. As such, modifications on which to focus include (but are not limited to) avoidance of salt and animal protein overconsumption and increasing intake of K^+^-containing bases and base precursors as well as fiber [2,104,105,106]. Most fruits and vegetables are rich in these elements. Available dietary recommendations and guidelines—although some are doctrine-based and lacking in rigorous human interventional and long-term study—are generally based on the pathophysiology of chronic disease processes and physiological capacity of the human body [2,107,108,109]. Adherence to the guidelines should be advocated. A multi-pronged approach aiming to improve all elements of lifestyle will likely be effective [110].

## 6. Summary

Notwithstanding a flexible homeostatic regulatory ability to efficiently respond and adapt to dynamic fluctuations of intrinsic (different stages of life phases and aging) and environmental (diet and living conditions) factors, the human body is, however through an evolutionary process, more acclimatized to a diet with abundant un-refined plant-based foods and constrained salt and animal meats. The modern diet, for the most part, is accepted as a norm when, in fact, it stands in contrast to the human body biology and physiology. It requires a sustained homeostatic regulation, operating at its extreme regulatory boundary, contributing to a multitude of metabolic abnormalities (glucocorticoid elevation, subclinical acidosis, muscle catabolism and bone demineralization) that are demonstratable even in healthy children and young adults. In the elderly, aging-related decline of organ function magnifies these maladaptive effects. It is not surprising that, by far, poor diet is the greatest contributor to the pandemic of chronic disease being experienced globally, especially in developed countries. Dietary and nutritional improvement represents an important disease-prevention strategy and has a great potential for improving public health. To paraphrase Aristotle: Nature does nothing without a purpose; virtue and excellences lie at the midpoint between two extremes (extreme deficiency and extreme excess). Promoting reasonable dietary moderation and avoidance of extremes, combined with physical activity and other elements of a healthy lifestyle [110], can bring harmony to the body’s biology and physiology, thus improving quality of life and minimizing risks for many modern era debilitating chronic diseases. This is of particular relevance to free-living omnivorous individuals with ready access to a virtually unlimited variety of foods.
